# Survival rate and outcomes of reverse total shoulder arthroplasty with a minimum ten-year follow-up using a trabecular metal implant

**DOI:** 10.1302/2633-1462.610.BJO-2025-0147.R1

**Published:** 2025-10-02

**Authors:** Shotaro Watanabe, Takuma Kaibara, Brian T. Feeley, Alan L. Zhang, Drew A. Lansdown, C Benjamin Ma

**Affiliations:** 1 Department of Orthopaedic Surgery, University of California, San Francisco, San Francisco, USA; 2 Department of Orthopaedic Surgery, Graduate School of Medical and Pharmaceutical Sciences, Chiba University, Chiba, Japan; 3 Center for Preventive Medical Sciences, Chiba University, Chiba, Japan; 4 Department of Orthopedic Surgery, Hokkaido University Graduate School of Medicine, Sapporo, Japan

**Keywords:** Reverse total shoulder arthroplasty, Trabecular metal, Long-term outcomes, Ten-year survival rate, reverse total shoulder arthroplasties, Scapular notching, American Shoulder and Elbow Surgeons (ASES) score, Pain score, Radiolucency, Glenoid, functional score, clinical outcomes, Kaplan-Meier survival analysis

## Abstract

**Aims:**

There are few reports of outcomes after reverse total shoulder arthroplasty (RTSA) with over ten years of follow-up. Further, there is a lack of reports on RTSA with trabecular metal (TM) implants with ten-year follow-up. We aim to assess the ten-year survival and minimum ten-year outcomes of TM-RTSA.

**Methods:**

All RTSA procedures were performed between October 2007 and July 2013 in a single institution. A consecutive series of 206 RTSAs in 194 patients were included in the Kaplan-Meier survival analysis using revision or removal for any reason as the endpoint. We also investigated the clinical and radiological outcomes at a minimum follow-up of ten years.

**Results:**

Out of 206 RTSAs, there were a total of 13 failures. The median time from surgery was 1.6 years (IQR 0.08 to 7.5). The five-year implant survival rate was 94.7% (95% CI 89.9 to 97.2; 102 RTSAs at risk), and the ten-year rate was 90.5% (95% CI 82.9 to 94.8; 62 RTSAs at risk). Minimum ten-year outcomes were available for 60 RTSAs, including 57 with ASES scores and 40 RTSAs with radiographs with a mean follow-up period of 11.3 years. The ASES score was a median pain score of 50 (IQR 45 to 50) and a median functional score of 36.7 (IQR 23.3 to 41.7) on the ipsilateral side. In radiological analyses for 40 RTSAs, scapular notching was observed in 31 RTSAs (77.5%) and classified as grade III or IV, as described by Sirveaux et al, in five RTSAs (12.5%). Glenoid radiolucency was observed in 11 RTSAs (27.5%) and loosening in three RTSAs (7.5%).

**Conclusion:**

TM RTSA demonstrated a high ten-year survival rate of 90.5%. Although radiological findings increased over time, clinical outcomes remained favourable.

Cite this article: *Bone Jt Open* 2025;6(10):1171–1178.

## Introduction

The modern reverse total shoulder arthroplasty (RTSA) was first introduced by Grammont.^1^ While there have been subsequent developments, the fundamental biomechanical concept of lowering and medializing the centre of rotation has remained a pivotal aspect of the procedure. With favourable postoperative results reported, the number of RTSA procedures has increased rapidly, and even in the medium-term results, improvements and maintenance of functional evaluation, including subjective evaluations and a range of motion (ROM), have been reported.^2-7^

There are still a small number of reports on long-term results. To date, only five papers have reported the outcomes of RTSA with a minimum follow-up of ten years.^8-12^ These studies include reports of good function being maintained more than ten years after RTSA, but with various complications, and implant survival rates ranging from 81% to 94%. However, as there are few reports on long-term results, it is not clear whether there are differences in postoperative outcomes, complications, and survival rates depending on the type of implant or surgical technique used.

A trabecular metal (TM) implant, porous-coated, was developed based on the original Grammont implant concept to achieve both initial stability and long-term results. By combining initial press-fit and screw fixation, the glenoid baseplate is fixed immediately, and the porous TM promotes bone ingrowth. The TM surface has been used extensively in the arthroplasty of the knee and hip joints, demonstrating favourable outcomes.^13,14^ Nevertheless, there are very few reports on TM implants in the RTSA.^15-19^ The longest follow-up period for these reports was a minimum of five years, and the long-term stability and outcomes remain unknown.

This study aims to report the results of RTSA using a single implant, porous-coated TM implant, from a single institution (University of California, USA) with a minimum follow-up of ten years.

## Methods

Patients provided informed consent and were enrolled in our prospective single-institution shoulder arthroplasty registry approved by our institutional review board. We retrospectively reviewed to identify the patients who underwent RTSAs performed between October 2007 and July 2013, that is, the patients who had been more than ten years post-surgery.

A consecutive series of 206 RTSAs (194 patients) met the inclusion criteria and were included in the survival analysis. The mean age at the time of surgery was 68.2 years (SD 11.1). Indications for RTSA were rotator cuff tear arthropathy, failed shoulder arthroplasty (hemiarthroplasty or TSA), osteoarthritis, fracture sequelae, acute fracture, and rheumatoid arthritis.

### Surgical technique

All 206 procedures were performed by one of two experienced surgeons (CBM, BTF) at a single institution with the same implant, a circumferential metaphyseal TM-coated humeral component and TM-backed glenosphere (on the baseplate and central peg) using the Zimmer trabecular metal Reverse Shoulder System (Zimmer Biomet, USA) which has a 143° neck-shaft angle and 2.5 mm centre of rotation offset. An elevated polyethylene liner of 7° was used, resulting in a neck-shaft angle of 150°. The deltopectoral approach was used in all cases. The baseplate was fixed to the glenoid neck with two superior and inferior locking screws. A 36 mm glenosphere was impacted on to the base plate. The humeral component was fixed using the cement technique for all cases. The tendon of the long head of the biceps, if present, was excised and a tenodesis was performed to the short head of the biceps. After the surgery, the arm was supported in a sling for six weeks postoperatively. Six weeks after surgery, supervised physical therapy without loading of the arm was started. Three months after surgery, physical therapy with loading was gradually allowed. Return to full activities without restrictions was commenced at six months.

### Clinical and radiological evaluations

The American Shoulder and Elbow Surgeons (ASES) score,^20^ which includes a pain score and a functional score on the ipsilateral and contralateral side, as patient-reported outcome measures (anterior elevation (AE), abduction (ABD), external rotation (ER), and internal rotation (IR)), and radiological evaluation were investigated. The radiographs were reviewed independently by two authors (SW, TK). In cases where the two independent evaluations did not agree, the grade was determined based on the discussions of the two and the opinion of the senior author (CBM). For grading scapular notching, the method described by Sirveaux et al^21^ was used. Radiolucency was defined as having a 2 mm or more space between bone and implant or cement. Glenoid radiolucency was evaluated in four zones as follows: 1) superior baseplate; 2) inferior baseplate (regardless of scapular notching, if present); 3) central pillar; and 4) screws.^22^ Migration was independently investigated and classified as positive when the baseplate location was significantly different from the previous location. Humerus radiolucency was also evaluated in seven zones described by Boileau et al.^22^ For both glenoid and humerus radiolucency, the presence of radiolucency in three or more zones was defined as loosening. Lateralization shoulder angles (LSA) and distalization shoulder angle (DSA) were measured using the method described by Boutsiadis et al.^23^

### Patient characteristics and outcomes at a minimum of ten years follow-up

**Fig. 1:**
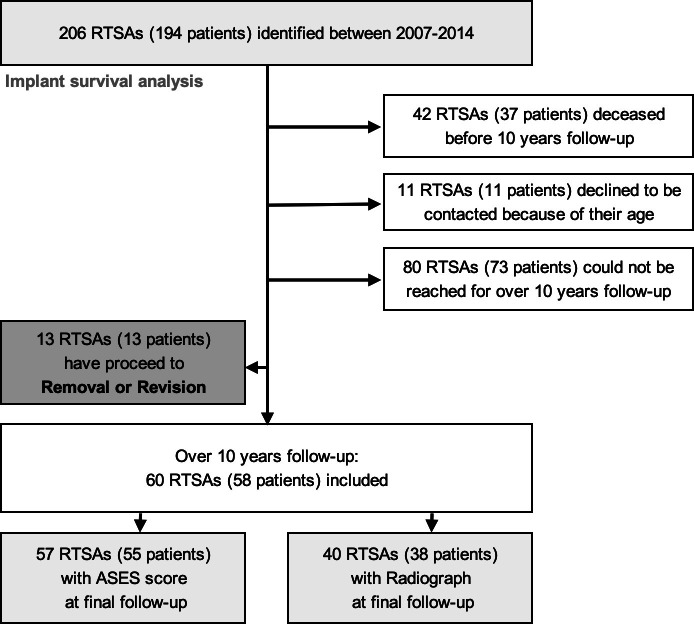
Flow diagram to identify the study cohort. RTSA, reverse total shoulder arthroplasty.

Out of 206 RTSAs (168 patients), 42 RTSAs (37 patients, 20.0%) were confirmed to be deceased before ten years post-surgery. Additionally, 80 RTSAs (73 patients, 43.5%) could not be contacted, and 11 patients declined any contact for research due to advanced age. Therefore, 60 RTSAs (58 patients) were available for follow-up beyond ten years, including 57 RTSAs (55 patients, 32.7%) with clinical outcomes and 40 RTSAs (38 patients, 22.6%) with radiological outcomes (Figure 1). The mean follow-up period was 11.3 years (SD 1.34). The demographic data of the 60 RTSAs (58 patients) is shown in Table I.

**Table I.** Demographic details of cases with a minimum of ten years follow-up.

Variable (n = 60)	Data
**Sex, n (%)**	
Female	27 (45)
Male	33 (55)
**Side, n (%)**	
Left	29 (48.3)
Right	31 (51.7)
Median age, yrs (IQR)	63.5 (56.5 to 72)
Median BMI, kg/m^2^ (IQR)	27.7 (24.1 to 31.5)
**Smoking, n (%)**	
No	28 (46.7)
Former smoker	28 (46.7)
Yes	4 (6.7)
**Diagnosis, n (%)**	
Rotator cuff tear arthropathy	26 (43.3)
Failed shoulder arthroplasty	16 (26.7)
Osteoarthritis	6 (10)
Fracture sequelae	5 (8.3)
Acute fracture	5 (8.3)
Rheumatoid arthritis	2 (3.3)
Procedure	54 (100)
Trabecular metal TM reverse shoulder, n (%)	60 (100)
Glenosphere size 36 mm, n (%)	60 (100)
Humeral cemented, (%)	60 (100)
Median operation time, mins (IQR)	99 (88 to 119)

### Statistical analysis

The survival rate was calculated by Kaplan-Meier survival analysis using revision or removal for any reason as the endpoint. For cases that died or lost to follow-up, the survival time up to the last follow-up was used. ASES score and ROM were compared preoperatively and postoperatively with Wilcoxon signed-rank test and among the indications with Kruskal-Wallis test. ASES score was compared between cases with or without radiolucency loosening, with and without scapular notching, and with and without scapular notching grade III or IV using Mann-Whitney test. LSA and DSA were also compared according to scapular notching with Mann-Whitney test. Spearman test was used to access the correlations between ASES score and LSA and DSA. All statistical analyses were conducted using Stata v. 18.5 (StataCorp, USA), with a significance level determined to be a p-value < 0.05.

## Results

### TM-RTSA survival

**Fig. 2:**
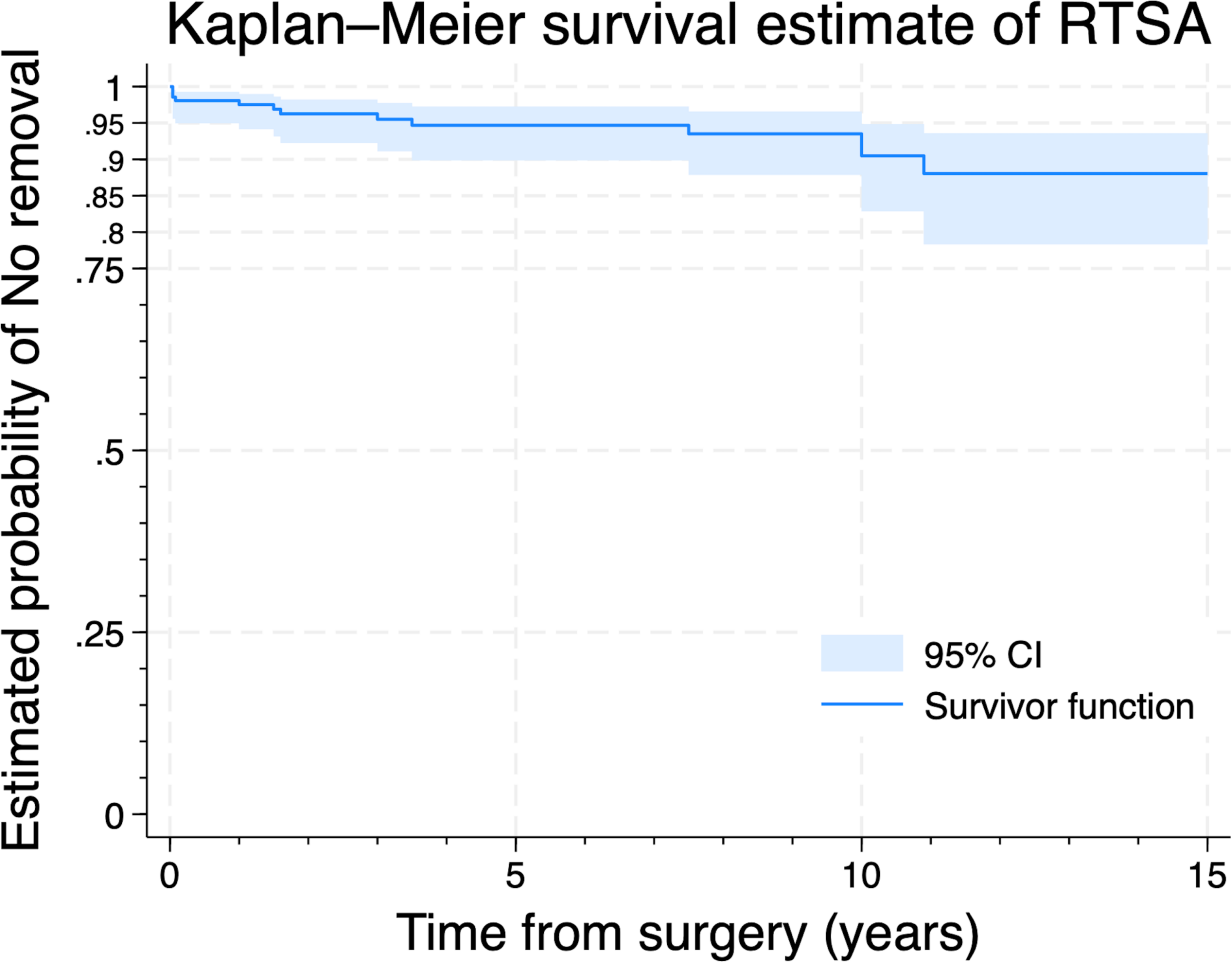
The implant survival rate of trabecular metal-reverse total shoulder arthroplasty (TM-RTSA).

Ten RTSAs were revised, and three were removed. The median time from surgery to revision or removal was 1.6 years (IQR 0.08 to 7.5). Kaplan–Meier survival analysis revealed that the five-year implant survival rate was 94.7% (95% CI 89.9 to 97.2; 102 RTSAs at risk), and the ten-year rate was 90.5% (95% CI 82.9 to 94.8; 62 RTSAs at risk) (Figure 2 and Table II). The reasons for revision or removal were infection in seven cases, dislocation in three cases, and glenoid component displacement in two cases, which were caused by trauma and delirium, and instability in one case (Table III).

**Table II.** The survival rate and number at risk of reverse total shoulder arthroplasties on yearly intervals.

Time, yrs*	At risk†	Fail‡	Survival rate	SE	95% CI	
1	177	5	0.9752	0.011	0.9414 to 0.9896	
2	152	2	0.9626	0.014	0.9226 to 0.9821	
3	129	1	0.9551	0.0158	0.9113 to 0.9775	
4	114	1	0.9468	0.0177	0.8987 to 0.9724	
5	102	0	0.9468	0.0177	0.8987 to 0.9724	
6	94	0	0.9468	0.0177	0.8987 to 0.9724	
7	91	0	0.9468	0.0177	0.8987 to 0.9724	
8	80	1	0.9351	0.021	0.8788 to 0.9658	
9	69	0	0.9351	0.021	0.8788 to 0.9658	
10	62	2	0.9049	0.0292	0.8285 to 0.9483	
11	36	1	0.8805	0.0373	0.7833 to 0.9358	
12	20	0	0.8805	0.0373	0.7833 to 0.9358	
13	12	0	0.8805	0.0373	0.7833 to 0.9358	
14	5	0	0.8805	0.0373	0.7833 to 0.9358	
15	1	0	0.8805	0.0373	0.7833 to 0.9358	

* Follow-up time in years since the surgery.

† Number of reverse total shoulder arthroplasties which were still under follow-up and had not experienced implant failure at the beginning of each interval.

‡ Number of reverse total shoulder arthroplasties which experienced implant failure within the interval, defined as either implant revision or removal.

SE, standard error.

**Table III.** Revision or removal cases.

Case	Age at surgery, yrs	Sex	Primary surgery diagnosis	Time to revision or removal, yrs	Reason	Procedures
1	55	Male	Nonunion after fracture	0.04	Dislocation	Liner change
2	56	Male	Nonunion after fracture	0.04	Dislocation	Liner change
3	53	Male	RCT arthropathy	0.04	Dislocation	Liner change
4	71	Male	RCT arthropathy	0.08	Glenoid component displacement	Conversion to hemiarthroplasty
5	84	Female	RCT arthropathy	1	Glenoid component displacement	Glenoid revision
6	81	Male	Osteoarthritis	1.5	Infection	Two-stage revision
7	69	Male	RCT arthropathy	1.6	Infection	Two-stage revision
8	70	Male	Failed shoulder arthroplasty	3	Infection	Two-stage revision
9	66	Female	Failed shoulder arthroplasty	3.5	Infection	Removal
10	44	Male	Failed shoulder arthroplasty	7.5	Infection	Two-stage revision, infection, removal
11	48	Male	RCT arthropathy	10	Infection	Two-stage revision
12	58	Female	Failed shoulder arthroplasty	10	Instability	Liner change
13	67	Male	RCT arthropathy	10.9	Infection	Two-stage revision

RCT, rotator cuff tear.

The ASES score at the final follow-up (n = 57) was a median pain score of 50 (IQR 45 to 50) and a median functional score of 36.7 (IQR 23.3 to 41.7) on the ipsilateral side. Comparing the preoperative and the final follow-up score in 37 RTSAs for which the preoperative data were available, the pain score and the functional score on the ipsilateral side demonstrated a statistically significant improvement (p < 0.001), whereas no such difference was observed in the functional score on the contralateral side (p = 0.222) (Table IV). The median ROM in 26 RTSAs with over ten years of ROM data was 160° (IQR 140° to 170°) for AE, 160° (IQR 140° to 170°) for ABD, and 37.5° (IQR 30° to 50°) for ER, respectively. The median level reached by IR was L2 (IQR T12 - Hip). Postoperative ROM improved from preoperative significantly except for IR (Table V). The result in each indication was shown in Table VI. There are no significant differences among indications in ASES score, AE, ABD, ER, and IR (p = 0.487, 0.514, 0.551, 0.791, 0.774, respectively).

**Table IV.** Comparison in American Shoulder and Elbow Surgeons score between preoperatively and a minimum of ten years postoperatively.

Score	Preoperative (n = 37)	Postoperative (n = 57)	p-value*
Median pain score (IQR)†	20 (10 to 40)	50 (45 to 50)	< 0.001
**Median function score (IQR)**			
Ipsilateral	18.3 (8.3 to 25)	36.7 (23.3 to 41.7)	< 0.001
Contralateral	43.3 (35 to 50)	43.3 (28.3 to 48.3)	0.222
**Median ASES (composite) score (IQR)** ‡			
Ipsilateral	38.3 (23.3 to 60)	85 (70 to 91.7)	< 0.001
Contralateral	58.3 (48.3 to 100)	88.3 (70 to 96.7)	< 0.001

* Wilcoxon signed-rank test.

† Pain score is not divided by sides.

‡ Composite score = pain score + function score. The contralateral side had a higher ASES score than the ipsilateral side preoperatively and even postoperatively (p < 0.001, p = 0.016, respectively).

ASES, American Shoulder and Elbow Surgeons.

**Table V.** Comparison in range of motion between preoperatively and a minimum of ten years postoperatively.

Variable	Preoperative (n = 47), median (IQR)	Postoperative (n = 26), median (IQR)	p-value*
Anterior elevation	90 (55 to 120)	160 (140 to 170)	0.002
Abduction	85 (50 to 110)	160 (140 to 170)	0.004
External rotation	30 (20 to 40)	37.5 (30 to 50)	0.044
Internal rotation	L5 (L1 - Hip)	L2 (T12 - Hip)	0.555

* Wilcoxon signed-rank test.

**Table VI.** American Shoulder and Elbow Surgeons score and range of motion at a minimum of ten years of final follow-up by indications.

Indication	n (%)	ASES score	AE	ABD	ER	IR
Rotator cuff tear arthropathy	26 (45.6)	86.7 (73.3 to 91.7)	165 (150 to 170)	160 (140 to 170)	42.5 (30 to 60)	L3 (T12-L5)
Failed shoulder arthroplasty	15 (26.3)	81.7 (53.3 to 88.3)	150 (130 to 165)	150 (110 to 170)	32.5 (30 to 40)	L2/3 (T12-Hip)
Osteoarthritis	6 (10.5)	81.7 (56.7 to 96.7)	160 (140 to 160)	150 (140 to 160)	42.5 (30 to 50)	L4/5 (L1-L5)
Fracture sequelae	5 (8.8)	85.0 (73.3 to 85.0)	160 (140 to 170)	160 (140 to 170)	40 (20 to 50)	L1 (T12-L2)
Acute fracture	3 (5.3)	96.7 (78.3 to 98.3)	160 (120 to 170)	160 (110 to 170)	30 (30 to 40)	T12 (T12-T12)
Rheumatoid arthritis	2 (3.5)	79.2 (76.7 to 81.7)	140 (120 to 160)	125 (90 to 160)	35 (20 to 50)	T12/L1 (T10-L3)

ABD, abduction; AE, anterior elevation; ASES, American Shoulder and Elbow Surgeons; ER, external rotation; IR, internal rotation.

**Fig. 3:**
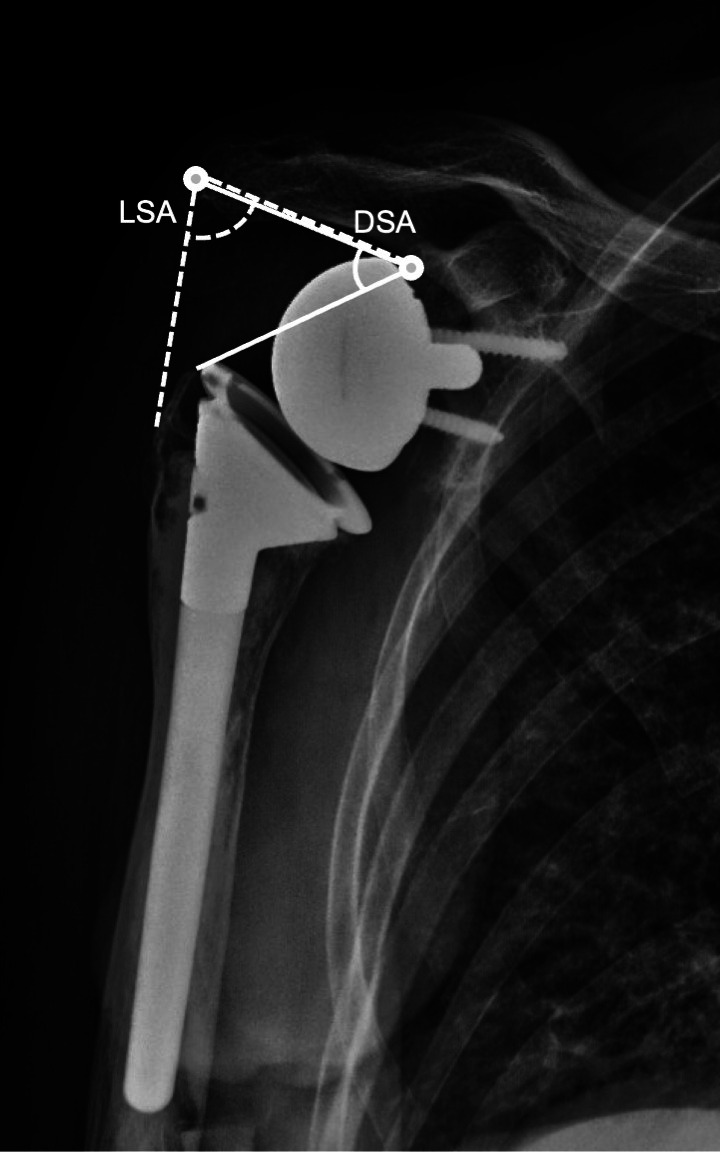
A case with scapular notching grade IV. LSA, lateralization shoulder angle; DSA, distalization shoulder angle.

In radiological analyses, as shown in Table VII, scapular notching was observed in 31 shoulders (77.5%) and classified as grade III or IV in five shoulders (12.5%) (Figure 3). Glenoid radiolucency was observed in 11 shoulders (27.5%) and loosening in three shoulders (7.5%), indicating two migration cases. Humerus radiolucency was observed in 16 shoulders (40.0%) and loosening in three shoulders (7.5%). The median LSA was 82.5° (IQR 75° to 86°) and the median DSA was 51.5° (IQR 41° to 57°). Three glenoid loosening cases, two of which had indications from 'failed shoulder arthroplasty' and one from 'fracture sequelae', had lower ASES scores than the others (p = 0.0103). Three humeral loosening cases, two of which had indications from 'failed shoulder arthroplasty' and one from 'fracture sequelae', had no significant difference (p = 0.535).

**Table VII.** Radiological findings at a minimum of ten years of final follow-up.

Radiological findings (n = 40)			
**Scapular notching, n (%)**		**Glenoid radiolucency, n (%)**	
None	9 (22.5)	None	29 (57.5)
Grade I	22 (55)	One location	6 (15)
Grade II	4 (10)	Two locations	2 (5)
Grade III	0 (0)	Loosening	3 (7.5)
Grade IV	5 (12.5)	**Location of radiolucency, n (%)**	
		Upper baseplate	7 (17.5)
**Humerus radiolucency, n (%)**		Lower baseplate	3 (7.5)
None	24 (60)	Peg or screw	2(5)
One location	7 (17.5)	Migration	2 (5)
Two locations	6 (15)		
Loosening	3 (7.5)	LSA, median (IQR)	82.5° (75° to 86°)
		DSA, median (IQR)	51.5° (41° to 57°)

DSA, distalization shoulder angle; LSA, lateralization shoulder angle.

There was no significant difference in ASES scores between cases with and without scapular notching (p = 0.88) in 37 cases for which data were available. However, patients with grade III or IV scapular notching had lower ASES scores than cases with lower grades (p = 0.024). The relationships between scapular notching and LSA and DSA were compared in 35 cases, excluding cases in which the humeral tuberosity was absent and could not be measured. No significant difference was observed in LSA and DSA between cases with and without scapular notching (p = 0.33, 0.44, respectively). However, DSA was larger in cases without scapular notching grade III or IV than in cases with those grades (p = 0.023), while there are no differences in LSA between those with and without scapular notching grade III or IV (p = 0.060). The relationships between ASES score and LSA and DSA were examined in the 32 cases for which data were available. However, no significant correlation was found (p = 0.436, 0.615, respectively).

## Discussion

### Implant survival

The five-year implant survival rate of 94.7% observed in this study is comparable to previous reports,^2,7,10,12,17,18^ and the ten-year survival rate of 90.5% is also similarly favourable when compared to previous reports using other implants with greater than ten years of follow-up.^6,8,10-12,24,25^ Ten-year survival rates varied from 81% to 94%.^8,10-12^ Chelli et al^6^ reported 80.9% for revision arthroplasty for failed arthroplasty. The longest reported survival rate for TM-RTSA was 94.8% at seven years (35 RTSAs) in a study with a minimum five-year follow-up.^18^ The seven-year survival rate in this study was 94.7% (91 RTSAs), showing very similar results, and this study reported a subsequent ten-year survival rate of 90.5% (62 RTSs). In this study when using a cementless TM baseplate, there were two cases of glenoid component displacement. Both cases occurred in the early postoperative period. One of the patients had postoperative delirium for several days and exhibited violent behaviour, necessitating physical restraint. The initial postoperative radiograph revealed dislocation, and the baseplate was removed and converted to a hemiarthroplasty. The other case, which failed at one year, had repeated falls from around three months postoperatively, and the patient’s dislocation progressed gradually, resulting in revision at one year. Thus, it is possible that strong external forces applied early, before initial stabilization is complete, may cause a glenoid component displacement, however, it was revealed that good long-term fixation would be achieved after initial stabilization was complete.

### ASES score improvement

In a review of reports with at least two years of follow-up, the ASES score has been reported to have improved from 37 preoperatively to 79 postoperatively.^26^ The ASES score was also reported to improve from 31 preoperatively to 70 at an average of 6.8 years,^2^ and other reports have shown an improvement from 35 preoperatively to 74 at the last follow-up at a minimum of ten years.^9^ Tashjian et al^27^ reported a 21-point improvement in the ASES score for minimal clinical importance difference (MCID) in shoulder joint arthroplasty. In this study, the ASES score improved from 38.3 preoperatively to 85 at an average of 11 years with a minimum ten-year follow-up. The preoperative scores were similar to previous reports, and the postoperative reports remained favourable despite ten years postoperatively, maintaining an improvement over the MCID of 21 points.

### Radiological outcomes

Scapular notching is one of the major radiological findings of RTSA. In a systematic review by Ernstbrunner et al^28^ it was reported that at 10 years postoperatively, 42% of RTSA had grade III or IV scapular notching. On reports using TM implants, in a study conducted by Theivendran et al,^17^ scapular notching was observed in 49.6% of cases with a minimum two-year follow-up period, while grade III or IV notching was identified in 0.8% of cases. Kankanalu et al^18^ used the same cohort and reported that scapular notching was present in 63.2% of cases with a minimum five-year follow-up, and that grade III or IV was present in 4% of cases. In our study, scapular notching was present in 77.5% of cases, but only 12.5% of cases had grade III or IV. Our results demonstrated the overall increase in notching grade over time since the five-year report. Nevertheless, the findings of TM baseplates were more favourable when compared to the previous results, which showed a 42% incidence of grade III or IV at ten years.^28^ The clinical significance of scapular notching remains to be elucidated. However, Favard et al^25^ have reported an increase in grades III and IV over time, which did not result in any effect on the Constant score. In contrast, Sirveaux et al^21^ reported that scapular notching greater than III affected the Constant score. The present study also demonstrated that scapular notching grade III or IV had an impact on the observed clinical score, the ASES score. Further research will be needed on this topic in the future.

The optimal angle range for increased anterior elevation and external rotation was from 40° to 65° for DSA and from 75° to 95° for LSA, as reported by Achilleas et al.^23^ These angles in this study were generally within this range, while DSA was larger in cases without scapular notching grade III or IV than in cases with those grades. However, there was no effect of these angles on the ASES score. Severe scapular notching affects the clinical score, while a small DSA alone does not affect the clinical outcome. A small DSA and a combination of other factors may reduce the clinical outcome when it leads to severe scapular notching.

We acknowledge that this study has limitations. First, our follow-up rate is low. This study was a ten-year follow-up of arthroplasty, and the patients were relatively elderly (the mean age was 68.2 years). We have 37 patients that were confirmed to be deceased and 73 patients we were unable to be reached despite different modes of contact. A number of these patients may be a result of death or non-response due to age. As for deceased cases, past RTSA follow-up reports over ten years have reported a death rate ranged from 30% to 50%,^8-12^ but in this study, only 42 cases (19%) were confirmed deceased. It is highly probable that well over half of the 73 patients lost to follow-up are also deceased, assuming a similar mortality rate of 30% to 50% as reported in other literature. This would suggest that 103 to 144 cases in 206 cases are likely still alive at ten years, and with 73 cases successfully followed up, the expected follow-up rate could be between 50.1% and 70.1%. This follow-up rate might be considered acceptable for a ten-year follow-up study involving elderly patients.

Second, all radiological evaluations were performed by radiography and not by CT, which can be more sensitive. This is an unavoidable limitation because this is a retrospective database study and postoperative CT is not part of our routine postoperative practice. Finally, it should be noted that this study was a single implant, single institution, two-surgeon cohort, making it difficult to generalize to the other RTSA designs or practice. However, the uniformity of implantation is a strength in terms of the reporting of implant results.

In conclusion, we present the outcomes of the RTSA using a TM implant from a single institution with a minimum follow-up of ten years and report an implant survival rate of 90.5% at ten years. At the final mean follow-up of 11.3 years, radiological findings increased while clinical outcomes remained to be favourable.

**Take home message**

- This is the first report to present a ten-year survival rate of reverse total shoulder arthroplasty (RTSA) using the trabecular metal (TM) implant and the clinical and radiological outcomes with a minimum ten-year follow-up.

- Consistent with previous TM-RTSA reports, a high incidence of radiological scapular notching was observed in this study at more than ten years of follow-up, which was more advanced than at five years in a previous report.

## Data Availability

The data that support the findings for this study are available to other researchers from the corresponding author upon reasonable request.
